# Metabarcoding of larval guts reveals diet diversity in native apex predators—the yellowjacket wasps *Vespula vulgaris* and* Vespula germanica*

**DOI:** 10.1007/s00040-025-01058-6

**Published:** 2025-08-21

**Authors:** Rosie Drinkwater, Georgia Law, Ben Bellekom, Robert L. Brown, William O.C. Symondson, Elizabeth L. Clare, Seirian Sumner

**Affiliations:** 1 School of Biological and Behavioural Sciences, Queen Mary University of London, London, E1 4NS UK; 2 Present Address: Palaeogenomics Group, Faculty of Veterinary Medicine, Ludwig Maximilian University Munich, Munich, 80539 Germany; 3 Centre for Biodiversity and Environment Research, Dept of Genetics, Evolution and Environment, University College London, Gower Street, London, WC1E 6BT UK; 4 Present Address: Department of Infectious Disease Epidemiology School of Public Health, Imperial College London, London, W12 0BZ, UK; 5 Manaaki Whenua-Landcare Research, P.O. Box 69040, Lincoln, 7640 New Zealand; 6 Cardiff School of Biosciences, Cardiff University, P.O. Box 915, Cardiff, CF10 3TL UK; 7 Present Address: Department of Biology, York University Toronto, Toronto, ON Canada M3J 1P3

**Keywords:** Wasps, *Vespula* spp., Diet analysis, Metabarcoding, Field sampling

## Abstract

Aculeate wasps are apex predators and play an important role in regulating arthropod populations. Determining what they hunt and how their diet varies with species ecology, geography and demography is essential to value their contributions as pest controllers in natural and human-modified ecosystems. There are many practical advantages in the study of social wasp diets. As central place foragers, hundreds of foragers bring prey back to the same location (the nest) every day; this means wasp diets can be explored by identifying taxa in prey balls collected directly from foragers arriving at the nest. A second advantage is that the colonies tend to contain large numbers of brood: prey can be identified from the gut contents of larvae or from the faeces that remain after metamorphosis. Morphological identification of insect remnants found in prey balls has provided insights into wasp diets but this approach is time-consuming and requires expert taxonomists. Metabarcoding is a more efficient method for describing insect diets; it has been used to identify prey of social wasps by sequencing prey balls, larvae guts and larval faeces. However, because of the ecological impact, data on social wasp diets are currently heavily skewed towards invasive populations. We lack comprehensive studies documenting what social wasps hunt in their native habitats. We use DNA metabarcoding to analyse the prey in over 500 wasp larvae guts from 14 nests of *Vespula* spp, sampled in their native range, across southern England. We describe the diverse diets of these insects, with the detection of 12 arthropod Orders in the guts of 554 larvae, and explore how diet varies across different locations. These data demonstrate the highly generalist predator role provided by *Vespula*, highlighting the key ecological roles they play as regulators of a broad range of arthropod taxa in native ecosystems and provide an important baseline for further exploring their ecological and economic value.

**Supplementary Information:**

The online version contains supplementary material available at 10.1007/s00040-025-01058-6.

## Introduction

*…“the practical result of destroying all the wasps on Sir T. Brisbane’s estate was, that in two years’ time the place was infested, like Egypt, with a plague of flies”.*

*Edward Latham Ormerod 1868*

Ormerod’s 1868 quote (Ormerod 1868) may be the first experimental evidence on the importance of wasps as regulators of arthropod populations. Despite this, 150 years later, we know remarkably little about the ecosystem services wasps offer, including as important pollinators and as nature’s pest controllers (Brock, Cini & Sumner 2021). Critical to this is a comprehensive understanding of wasp diets. The first scientific records of social wasp diets are from the early 1800s, by naturalists who intercepted foragers returning to their nests to retrieve prey balls of mangled invertebrates (Spradbery 1973); although social wasps kill and dismember their prey, body parts are often still intact enough to be identified, by expert taxonomists. Thanks to these painstaking dissections of prey balls, we have long known social wasps to be voracious hunters of a diverse range of invertebrates, including a wide range of pest species. These traditional approaches require taxonomic expertise for identification and are not necessarily scalable across large areas, given the specific timing required to intercept prey-bearing wasps. Advances in molecular techniques now make it possible to explore the diets of wasps more broadly and more efficiently (see references in Table 1 and Supplementary Data S1). Such studies are vital for understanding the impact of wasps in ecosystems whether as natural capital in their native regions (Kasper et al 2004; Jeon et al 2019) or as potential ecological threats in regions where they are invasive (Lester & Beggs 2019). In a world where we are increasingly concerned about the state of insect populations and the loss of their functions in ecosystems (Outhwaite et al. 2020), such studies have never been more timely.

**Table 1:** Summary of existing studies on wasp diet (full data given in Supplementary Data S1). There are no molecular analyses for Vespines sampled from native populations. Note that totals do not result in the sum of the columns, since some of the same species are included in both methods—molecular and morphological

Taxa	Method	No. of independent studies	No. of species	No. species studied in	
				Native range	Invasive range
All species	Molecular	8	8	2	7
	Morphology	10	12	9	6
	Total	17	14	10	7
Vespines	Molecular	4	4	0	4
	Morphology	9	10	8	4
	Total	12	10	8	4
Polistes	Molecular	6	4	2	3
	Morphology	2	2	1	2
	Total	7	4	2	3

To identify key knowledge gaps, we collated data from the literature on higher level taxonomic diversity of social wasp diets, tissues sampled (prey balls, larval guts, meconium), the identification methods (morphological or molecular), sample sizes and whether they were sampled from native or invasive populations (Supplementary Data S1). Social wasps comprise three subfamilies: the Vespinae (hornets and yellowjackets), Polistinae (paper wasps) and the Stenogastrinae (hover wasps). Prey records for Polistinae and Stenogastrinae are largely observational (and not included in Supplementary Data S1); an exception is the genus *Polistes,* where six molecular diet studies exist for four species. We focus here on the Vespine wasps because, whilst there are the highest number of individual diet studies for this family (12 studies, Table 1), covering four *Vespula* and six *Vespa* species, these are largely morphological and there is no molecular diet analysis for Vespine wasps in their native range (Table 1).

Collectively, Vespines are generalist hunters of arthropods and occasional scavengers of carrion. They are recorded hunting prey from diverse taxonomic groups; most records come from Insects (9 Orders) and Arachnids (4 Orders), but occasionally diets include Annelida, Isopoda, Gastropoda, Cephalopoda, mammals and birds. Only 4 of the 12 Vespine studies employ molecular methods (e.g. see Liu et al., 2020, Supplementary Data S1). This includes targeting COI from the meconium (a substance excreted by larvae into the bottom of cells when they pupate) for *V. vulgaris* (48 cells from 16 nests) and *V. germanica* (9 cells from 3 nests) in New Zealand where they are notoriously invasive (Schmack et al. 2021). Kasper et al. (2004) sequenced the 16S region from 44 prey balls of invasive *V. germanica* in Australia, and Wilson et al. (2009) applied Sanger sequencing to analyse both 16S and COI markers from 412 prey balls collected from 10 nests of *Vespula pensylvanica* from Hawaii*.* In the most recent molecular study, Stainton et al. (2023) applied metabarcoding with Nanopore (ONT) sequencing to the gut contents of 38 larvae from 5 nests of *Vespa velutina*, another invasive population of Vespine wasps in Southern UK and the Channel Islands. However, species accumulation curves were assessed for only the two most recent studies (Schmack et al 2021; Stainton et al 2023) and neither reached an asymptote (Supplementary Data S1), indicating that they were missing prey species. Most critically, all these studies are from invasive populations of Vespines which are likely to have altered diets from those in native populations. We have, therefore, identified a gap in the literature with respect to molecular analyses of diets for Vespines in native regions.

Here we provide the first molecular analyses on the diets of Vespine wasps in native populations. Using DNA metabarcoding of larval guts from 14 nests we: (1) describe the prey diversity of *Vespula vulgaris*; (2) compare this across different geographic locations; (3) compare potential prey resources to actual consumption for one focal colony of *V. vulgaris*; and (4) provide a preliminary prey list for *V. germanica* in their native range, from a single focal colony and compare with available prey, providing a baseline for future work. These data on the diets of native *Vespula* wasps take us one step closer to understanding the natural capital of these insects in native ecosystems.

## Methods

### Sample collection and larval dissections

Wasp nest sampling was conducted in situ at five sites in September–October 2016 and two sites in September–October 2018, with the resampling of one site at Silwood Park in both years (Supplementary Table S1; Supplementary Figure S1). Wasp nests were excavated from below ground on all sites whilst the colony was active. Once the top of the nest was uncovered, each layer of comb containing the larvae was removed and stored at −20 °C until larval dissections. In total, 554 larvae were sampled from 13 *Vespula vulgaris* colonies and 1 *Vespula germanica* colony. The gut of each larva was removed by dissection under a microscope. Phosphate-buffered saline (PBS) solution was applied to increase motility so that layers of larval tissue could be cleaned from the gut membrane. The gut was cleaned without rupturing the gut membrane and was transferred to 80% ethanol until DNA extraction.

### Molecular diet analysis

Dissected wasp guts were removed from ethanol, and gut contents were released into 180 μl of ATL buffer (Qiagen, UK), removing the gut lining to reduce contamination. DNA was extracted following the Qiagen DNeasy Blood and Tissue kit (Qiagen, UK), with modifications  to the manufacturers protocol including an extended digestion with proteinase K (approximately 12 h at 56 C), the centrifuge speed was increased to 16,000 g on addition of buffer AW2 and elution buffer volume was reduced to 35 μL. Four blank extractions were conducted, following the same protocol as the wasp gut contents.

From each gut extraction, we amplified a 314 bp fragment COI mitochondrial gene in a PCR reaction using the Beren–Luthien primer set, designed to capture arthropod DNA (Cuff et al. 2021). Each 15 μL reaction used 7.5 μL of Multiplex PCR mastermix (Qiagen, UK), 0.25 μL of each primer (10 μM), 5 μL H_2_O and 2 μL template DNA. Negative and positive controls were included in PCR reactions and later sequencing. The thermal cycling protocol was as follows: 95 °C for 15 min, 34 cycles of 94 °C for 40 s, 40 °C for 1 min, 72 °C for 30 s, followed by a final extension of 72 °C for 5 min. Amplicon sequencing was conducted by the Bart’s and the London Genome Centre, London, UK, alongside samples from an unrelated project. All PCR products were processed on site including size selection using Ampure beads (Beckman Coulter, Brea, CA, USA), quantification, quality control (QC) and normalisation using a Qubit (Invitrogen, Carlsbad, CA, USA), and DNA D100 Tape station (Agilent). Each amplicon was indexed and sequenced on an Ilumina MiSeq (2 × 250 bp) using V2 chemistry following Hodgkiss et al. (2022).

### Arthropod field sampling

At one site, within 100 m of the colonies at Regent’s Park, we deployed four arthropod sampling techniques to sample the range of prey items available to *Vespula sp*. and compared these morphotypes to the identified dietary items. Between 20 and 27th September 2018, we conducted the arthropod survey using sweep netting (*n* = 8), beating (*n* = 8), pan traps (*n* = 4) and a malaise trap (*n* = 1). These methods target different taxa, so by combining them, a broad taxonomic range of potential arthropod prey could be sampled (Yi et al., 2012). Full details are included in the Supplementary Methods. The individual arthropods from each method were identified to Order, detections across all methods were merged and the percentage of detections was calculated which can then be compared with the percentage of Orders detected from wasp larval guts.

### Generating mock communities

The specimens collected during the field sampling were then used to create positive controls and to establish appropriate filtering parameters for our data. DNA was extracted from individual arthropods (following the same method as the larvae above) and pooled together to build two DNA mock communities containing a mix of potential prey targets from the field collected arthropod samples. These communities included seven Orders (Araneae, Coleoptera, Diptera, Hemiptera, Hymenoptera, Lepidoptera, Orthoptera). The mock communities were sequenced as well as the individual DNA extracts from the arthropods along with larval samples (see above).

From the sequencing results we checked we had successfully identified each individual arthropod before we analysed the mock communities to establish the required filtering parameters for the metabarcoding data. This included the removal of arthropod IDs with fewer than 200, 400 or 1000 reads, to establish the baseline by which we could recover true identifications in our dietary dataset (Supplementary Methods and Table S2). Between the two communities, at 1000 reads, we lost between 2–4 Orders; at 400 reads, we lost 1–3 and at 200 reads, we lost 0–2. As a result, we employed a filter of 200 reads which maximised recovery for both mock communities whilst minimising contamination.

### Taxonomic assignment

The resulting sequence data were assembled using USEARCH to pair the forward and reverse reads (Edgar 2010). Then the consensus sequences were processed using the mBRAVE online platform (Ratnasingham 2019), a sister platform to the Barcode of Life Data system (Ratnasingham & Hebert, 2007). Due to the degraded nature of DNA from gut samples, data of all quality were included but the primer sequences were removed from reads by length trimming at the 5’ and the 3’ end of the sequence. The ID distance threshold was set to 2%, so sequences that were 98% similar to the reference were assigned taxonomy. The filtered sequences were then compared sequentially to three of the BOLD system reference databases, and taxonomic identifications for each sample were downloaded for further analysis. The databases were (1) SYS-CRINSECTA for insects, (2) SYS-CRLNONARTHINVERT for non-arthropod invertebrates and (3) SYS-CRLNONINSECTARTH for non-insect arthropods. Based on the results of the mock community analysis, a sequence threshold was set for removing those assignments with less than 200 reads. Any predator DNA assignments (i.e. *Vespula* sp) were removed together with any spurious prey identifications (i.e. from non-UK species). The taxonomic assignments are presented in Supplementary Data S4, and were collapsed to Order and for analysis.

### Dietary diversity analysis

#### Diversity accumulation curves

First, we analysed the diversity of prey accumulated in the *V. vulgaris* guts at each of the nine sites, to compare sampling effort and identify any under-sampling. This resulted in six well-sampled sites (reaching an asymptote) which were used for the further analyses of alpha and community diversity (Supplementary Fig. 1). Accumulation curves were generated in “iNEXT” (Hsieh et al., 2016) in R studio (R Core Team, 2019).

#### Dietary diversity in *V. vulgaris* (six well-sampled sites) and comparison to available arthropods at Regent’s Park

To characterise the alpha diversity of the *V. vulgaris* diet, we calculated the amount of detections of arthropods in the guts from the six well-sampled sites. At the Regent’s Park site, we compared the percentage of detections made by the molecular vs. the field sampling.

#### Geographical variation in *V. vulgaris* diet (six well-sampled sites)

To investigate the spatial variation in diets from wasp nests across different sites, we used a PERMANOVA model on prey counts and Bray–Curtis dissimilarity matrix to analyse whether community composition varied by sampling location. These models were constrained to account for colonies within locations, using the default parameters (including 999 permutations) in “vegan” (Oksanen et al 2010) in R studio (R Core Team, 2019) with the following formula, which restricts the permutations to within the colonies at different sites:

We calculated site differences between groups using non-metric multidimensional scales (NMDS), based on the same Bray–Curtis dissimilarity matrix specifying three axes, with 500 max tries and all other defaults conducted using “vegan” (Oksanen et al 2010) in R studio (R Core Team, 2019).

#### Summary of *V. germanica* diet and comparison to available arthropods at Regent’s Park

We calculated the percentage contribution of each prey species in the *V. germanica* diet and compared this to the field sampling.

All graphics, for all analyses, were produced using the package “ggplot2” (Wickham & Chang 2008).

## Results and discussion

We generated 3,105,857 raw sequencing reads. After quality filtering steps, 404 detections corresponded to the predators (*Vespula* spp*.*) and were removed from further analysis. This left 1387 individual detections recorded from 554 larval gut samples: 1181 detections in the 474 V*. vulgaris* larval guts and 206 detections in the 80 V*. germanica* larval gut samples. These were all assigned to 213 unique taxon (Supplementary Data S4).

### Broadest diversity of arthropod diet for *V. vulgaris*

Diversity accumulation curves showed that three sites were severely under-sampled (Bristol, Parke Estate and Petworth House) and these were removed from further analysis. This removal only results in the loss of 17 samples and 36 detections (3%) for *V. vulgaris*. The diversity curves of the remaining six sites suggest that further sampling effort would not have increased diet diversity detected and all further analyses are based on these sites only (Supplementary Fig. S2). Our dataset is, therefore, the most complete catalogue of Vespine diets to date (cf data in Supplementary Data S1).

**Fig. 1:**
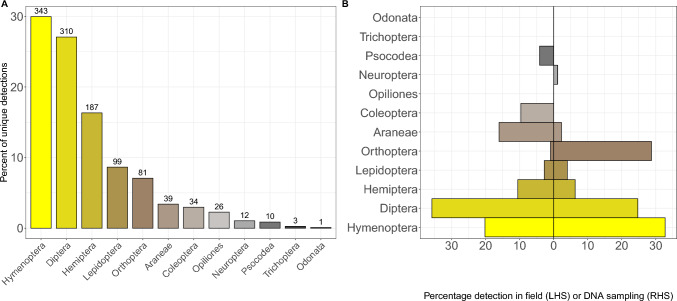
Arthropod diversity across *Vespula vulgaris* diets. **A** Percentage contribution of each diet taxa found across *V. vulgaris* samples at the six well-sampled sites. The number of detections for each Order are given on top of each bar. **B** Comparison between field sampling and OTU detections at one site. Percentage of arthropod Orders detected in the field sampling on the left side (LHS) and molecular analysis on the right side (RHS) of zero

When calculating alpha diversity across the 457 *V. vulgaris* larvae, from the 6 sites, there were 1145 detections from 12 arthropod Orders (Fig. 1A) and 86 families from the class Insecta and Arachnida (Supplementary Data S4). This diversity reflects the broadest range of arthropod taxa yet detected in social wasp diets (Supplementary Data S3). Whilst providing the first records of Trichoptera (caddisfly) in the diets of a social wasp, we found no evidence of one Order previously detected in Vespine diets within their native range (Dermaptera (earwigs) (Spradbery 1973; Archer 1977) see Review in Supplementary Dataset 1). Also of note, almost 60% of prey belonged to Hymenoptera (30%—4 families) and Diptera (27%—26 families) (Fig. 1A). Non-arthropod taxa were not detected since the primers used were designed to preferentially capture arthropod DNA (Cuff et al. 2021); other groups (e.g. vertebrates) may crop up in *Vespula* diets as they are also scavengers but arthropods remain the vast majority of their diet (see Supplementary Table 1). Explanations for the extraordinary breadth of diet captured by our data in comparison to the previous studies may be due to sampling methods (numbers of larvae analysed, and/or the wide geographical locations captured) or local/native ecology. However, quantitative comparisons across these datasets are not possible because there is no standardisation across studies with respect to location, species, sampling methods and depth (see Supplementary Data S1). Yet our data provide a much-needed baseline for these species in their native zone.

### *V. vulgaris* gut contents represent available potential arthropod prey

The arthropod field sampling at the Regent’s Park site resulted in 2870 individual arthropods, which were assigned to 218 morphotypes from 8 Orders (Supplementary Data S2). There was a high amount of overlap between the arthropod diversity in field samples and the guts sampled at the same site (Fig. 1B). The 174 unique hits from the Regents park gut samples included OTUs from 7 of the same 8 Orders found in the field sampling; Coleoptera was missing from these guts (although they were detected as prey at other sites) and Neuroptera was detected only in the guts (Fig. 1B). This comparison confirms that wasps have a highly generalist diet, taking the opportunity to hunt whatever arthropod prey is available. Representation of diet revealed by the two sampling methods differed for some Orders; this could reflect some diet preferences by the wasps, or that wasps may not sample the environment in the same way as Malaise traps. In addition, not all arthropods will be digested equally, so these discrepancies are likely to represent subsequent amplification bias, and for this reason, comparisons of gut contents with prey consumed can be challenging (Pompanon et al., 2012, Deagle et al. 2019).

### *V. vulgaris* diets vary with location but inter-colony differences are greater

**Fig. 2:**
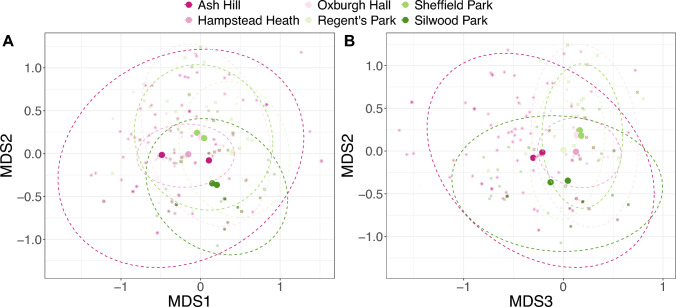
Community dietary diversity analysis limited to the six exhaustively sampled sites (6 sites; 10 colonies; 457 larvae). **A** NMDS axis 1 and 2 and** B** NMDS axes 2 and 3. The calculated stress value for the ordination is 0.1. Large solid circles show the centroid mean value per colony, whilst the dashed lines show the 95% ellipse

The results of the PERMANOVA revealed that diets were different at the different sites (R^2^ = 0.20, F = 23.64,Fig. 2), but this was not significant when accounting for colonies within location (Supplementary Table S3). This indicates that there is high heterogeneity between colonies even at the same location. Ash Hill wasp guts exhibited the most diverse diet and largest variance, with all other sites nested within it, whilst Hampstead Heath had the most limited dietary diversity and smallest group variance (Fig. 2). This is interesting since Hampstead Heath and Ash Hill are only 30 miles apart, but it should be noted that Hamstead Heath is in a highly urbanised area of Central London whilst Ash Hill is a more rural habitat.

### First diet characterisation reported for *V. germanica* from a native population

**Fig. 3:**
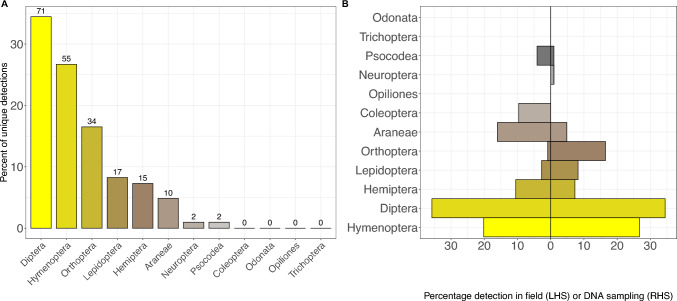
Arthropod diversity across *Vespula germanica* diets at Regent’s Park. **A** Percentage contribution of each diet taxa found across *V. germanica* samples at the one sampled site—Regents Park, with the total number of detections per Order given on top of each bar. **B** Comparison between field sampling and OTU detections at one site. Percentage of arthropod Orders detected in the field sampling on the left side (LHS) and molecular analysis on the right side (RHS) of zero

At one site, Regent’s Park, we analysed 80 larval gut samples from a *V. germanica* nest providing the first assessment of the diet for this species in its native range. From these samples, we retrieved 206 arthropod detections from 8 Orders (Fig. 3A). Whilst *V. germanica* diets revealed a lower percentage of Hymenoptera and Orthoptera, there was a higher percentage of Diptera in the diet compared with *V. vulgaris* at the same site (Fig. 3B).

## Conclusion

Insects provide essential ecosystem services (Noriega et al., 2018). Those provided by aculeate wasps have been largely overlooked, compared to other similar taxa such as bees, flies, beetles and butterflies which are widely valued as pollinators, decomposers and predators (Sumner, Law & Cini 2018), and wasps are sorely understudied (Oi et al. 2024). Although we have records of what wasps were hunting dating back over 200 years, we lacked a comprehensive understanding of prey diversity and how this varies with prey availability, colony, location and species. Moreover, data were surprisingly limited despite these being some of the commonest and wide-spread insects encountered in their native regions. Our analyses begin to plug these gaps, using metabarcoding of larval guts for the two commonest Vespine wasps in Europe—*V. vulgaris* and *V. germanica*. We detected the widest diversity of arthropod prey reported to date for a vespine wasp. We show that the diet of the more common *V. vulgaris* largely reflects local prey diversity, and, whilst diet does vary somewhat with location, most variation in diet is explained by between-colony dietary variation. This is very interesting, as it suggests that there are colony-level preferences in prey choice. Previous work on *Polistes* paper wasps has suggested that foraging wasps can become ‘fixated’ on specific prey types (Gould & Jeanne 1984). This is a possible explanation for the high inter-colony differences we detected, irrespective of location.

Taken together, these findings suggest that wasps are generalist predators and are likely to play important roles in regulating arthropod populations, but that their actual impact will vary depending on the local habitat and species assemblages. These baseline data on the diets of native yellowjacket wasps provide an essential bedrock on which to build a much-needed understanding on the value of these insects as apex predators in diverse ecosystems.

## Supplementary Information

Below is the link to the electronic supplementary material.

Supplementary file1 (XLSX 87 KB)

Supplementary file2 (DOCX 374 KB)

## Data Availability

Genetic sequence dataset is available on figshare project 10.6084/m9.figshare.29315018.
