# Supplementary material for: Metabarcoding of larval guts reveals diet diversity in native apex predators—the yellowjacket wasps Vespula vulgaris and Vespula germanica
Source: Insectes Soc. 2025 Aug 21;73(1):37–44. doi: 10.1007/s00040-025-01058-6 (PMC12901276; doi:10.1007/s00040-025-01058-6)
Supplement: Supplementary file 2 — Supplementary file2 (DOCX 374 KB) [file 40_2025_1058_MOESM2_ESM.docx]

**Supplementary material for:**

**Metabarcoding of larval guts reveal diet diversity in native apex predators – the yellowjacket wasps *Vespula vulgaris and Vespula germanica***

1. **Supplementary methods**

**Arthropod field sampling**

At the Regents Park site we deployed the following methods between 20 - 27th September 2018, to sample arthropods:

1. A 30m sweep transect was set up in an area of long grass and sampled 8 times. Using a sweep net, the transect was walked slowly, sweeping once per meter, then the catch was transferred to a single tube (Spafford & Lortie, 2013, McGavin 2007).
2. A 50m beating transect was also set up through a wooded area and each tree along the transect was beaten sharply 3 times onto a beating sheet. After each beat, the contents of the sheet were transferred into a large butterfly net so that the arthropods could be prevented from flying or crawling away (McGavin 2007).

Arthropods from both transects were killed using ethyl acetate fumes and each transect was treated as an independent sample.

1. Pan traps (n= 4) consist of a stake that sits in the ground with holders for three coloured bowls (yellow, blue and white) that can be raised and lowered to be level with vegetation height (Carvell et al., 2016). Ten pan traps were split into two transects of 40m and set in an area of long grass for 4 days, with each trap set 10m apart. Following the Centre for Ecology and Hydrology’s protocol, a mix of 2l water with approx. 5ml detergent was used as a stock solution to fill each trap (Carvell et al., 2016, Spafford & Lortie, 2013). The bowls were filled ¼ full with the solution (approx. 100ml) (Carvell et al., 2016).
2. A malaise trap (n = 1) was erected on the field site following the manufacturer’s instructions, perpendicular to an arthropod flight path with the top end south-facing and left for 6 days with 80% ethanol in the sampling bottle.

All arthropod samples from each method were transferred to 80% Ethanol and stored in the fridge until identification. The specimens were identified to Order (McGavin 2001) and then within Order split into morphotypes (Supplementary Data S2). Considering all the methods together, the percentage of detections of each invertebrate order was calculated and compared with the percentage of orders detected from wasp guts.

**Mock community analysis and filtering thresholds**

We then analysed the pooled mock communities using a filtering threshold which included the removal of any ID with fewer than 200, 400 or 1000 reads (Supplementary table S1).

- *Mock community 1* had the most efficient data recovery, where at the strictest filtering level (minimum of 1000 reads) the Araneae and Hymenoptera were lost, with minimum 400 reads just the Hymenoptera were removed and with a minimum 200 reads all 7 orders were recovered without any false positive data (Supplementary Table S2).
- *Mock community 2* had a similar outcome but was less efficient where at 1000 reads additional to Araneae and Hymenoptera, Diptera and Lepidoptera were lost. At a 400 read minimum, Hymenoptera, Diptera and Lepidoptera were not recovered and at 200 filtered reads Diptera and Hymenoptera were not recovered.

The highest read count for a false positive (contamination or false positive ID was 17 reads). As a consequence we employed a filter of 200 reads which maximized recovery for both mock communities while minimizing contamination.

**Supplementary References:**

- Spafford, R. D., & Lortie, C. J. (2013). Sweeping beauty: is grassland arthropod community composition effectively estimated by sweep netting?. *Ecology and Evolution*, *3*(10), 3347-3358.
- McGavin, G. (2007). *Expedition field techniques: Insects and other terrestrial arthropods*. Geography Outdoors: The Centre Supporting Field Research, Exploration and Outdoor Learning.
- Carvell, C., et al. (2016). *Design and testing of a national pollinator and pollination monitoring framework: Final summary report to the Department for Environment, Food and Rural Affairs (Defra), Scottish Government and Welsh Government: Project WC1101*.
- McGavin, G. C. (2001). *Essential entomology: An order‑by‑order introduction* (pp. vi–318). Oxford University Press.

1. **Supplementary Tables**

| **Supplementary Table S1.** Summary of the 14 colonies sampled across nine locations in Southern England. The number of larval individuals for which OTUs could be recovered and identified to describe diet are shown. The two Regent’s Park nests were sampled from the same location from both species: *V. germanica* samples are highlighted in grey. The caste of individual larvae was determined for those sampled in 2018. Full data can be found broken down in Supplementary Data S3. | | | | |
| --- | --- | --- | --- | --- |
| Site | Species | Year | No. of Larvae | Total OTUs detected |
|  |  |  |  |  |
| Ash Hill | *V. vulgaris* | 2016 | 60 | 156 |
| Ash Hill | *V. vulgaris* | 2016 | 56 | 112 |
| Bristol | *V. vulgaris* | 2016 | 5 | 17 |
| Hampstead Heath | *V. vulgaris* | 2018 | 80 | 233 |
| Oxburgh Hall | *V. vulgaris* | 2016 | 41 | 88 |
| Oxburgh Hall | *V. vulgaris* | 2016 | 36 | 78 |
| Parke Estate | *V. vulgaris* | 2016 | 7 | 8 |
| Petworth house | *V. vulgaris* | 2016 | 5 | 11 |
| Regent's Park | *V. vulgaris* | 2018 | 60 | 174 |
| Regent's Park | *V. germanica* | 2018 | 80 | 206 |
| Sheffield Park | *V. vulgaris* | 2016 | 31 | 73 |
| Sheffield Park | *V. vulgaris* | 2016 | 30 | 75 |
| Silwood Park | *V. vulgaris* | 2016 | 27 | 74 |
| Silwood Park | *V. vulgaris* | 2018 | 36 | 84 |

| **Supplementary Table S2.** Mock community analysis of two communities (MC1 and MC2) at three filtering thresholds - where a minimum number of reads was required to assign the taxonomy.. A green box indicates that the expected order (i.e. the DNA that was inputted) was recovered with no false positives or contamination. | | | | | | |
| --- | --- | --- | --- | --- | --- | --- |
| **Order** | **Filter Threshold** | | | | | |
|  | 200 reads min | | 400 reads min | | 1000 reads min | |
|  | MC1 | MC2 | MC1 | MC2 | MC1 | MC2 |
| Araneae |  |  |  |  |  |  |
| Coleoptera |  |  |  |  |  |  |
| Diptera |  |  |  |  |  |  |
| Hemiptera |  |  |  |  |  |  |
| Hymenoptera |  |  |  |  |  |  |
| Lepidoptera |  |  |  |  |  |  |
| Orthoptera |  |  |  |  |  |  |

| **Supplementary Table S3.** PERMANOVA model results. Including the specified model structure, R2 and p-values for the (1) *V. vulgaris* diet comparison across locations model | | | | | |
| --- | --- | --- | --- | --- | --- |
| **Model Structure (1)** | | ***V. vulgaris* diet ~ Location / Colony, strata = Colony** | | | |
|  | **Df** | **SumOfSqs** | **R2** | **F** | **Pr(>F)** |
| **Location** | 5 | 17.74 | 0.20 | 23.64 | 1 |
| **Location: Colony** | 4 | 3.86 | 0.04 | 6.42 | 1 |
| **Residual** | 447 | 67.08 | 0.76 |  |  |
| **Total** | 456 | 88.67 | 1.00 |  |  |

1. **Supplementary Figure**

**Supplementary Figure
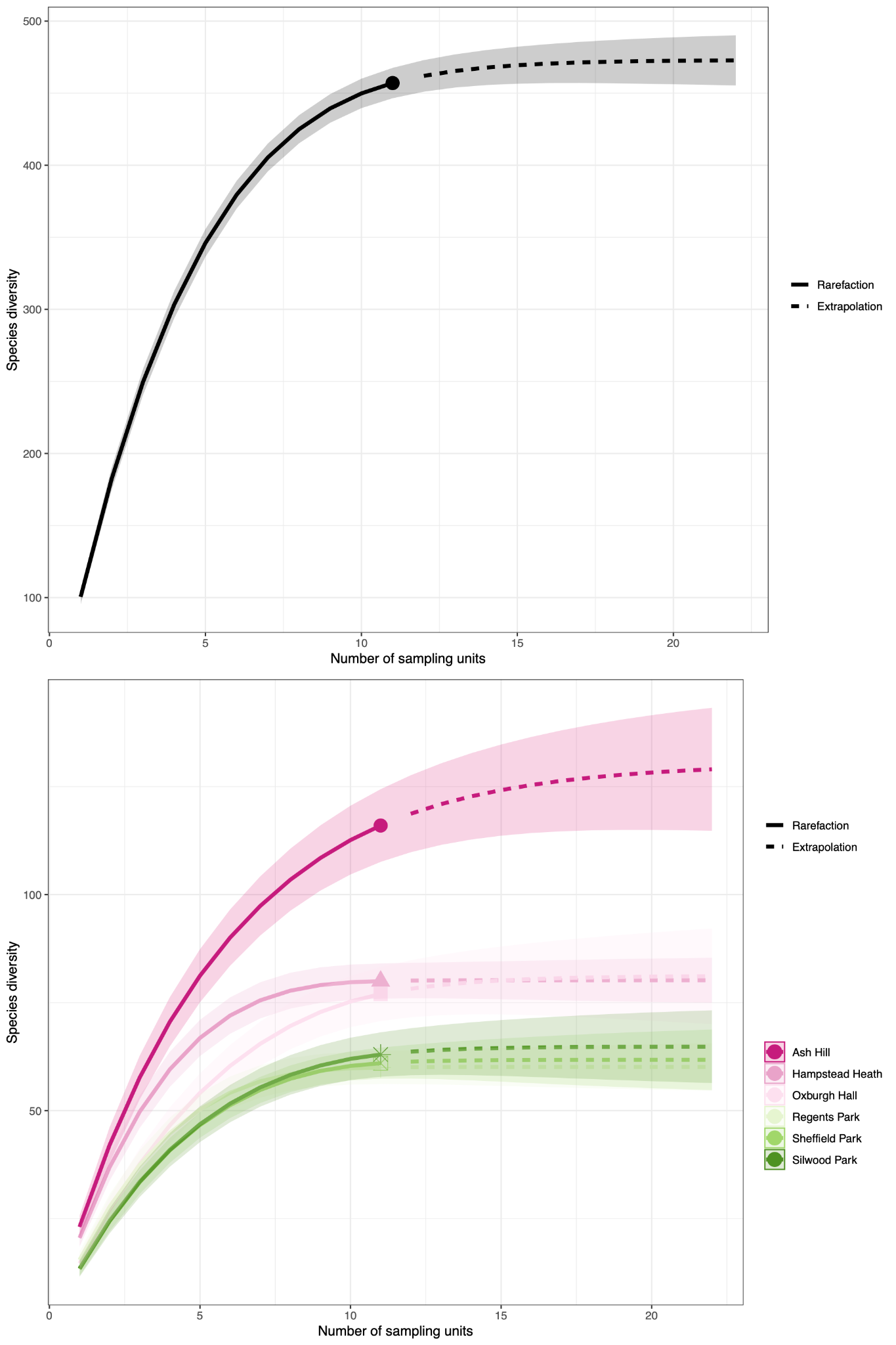
**

**S1. Species diversity accumulation curves for prey species in the diets of *V. vulgaris* based on OTUs.**

In the top graph all samples were combined from all sites. In the bottom graph we separated samples by site (without the three sites which are severely under sampled; Bristol, Parke Estate and Petworth House). The accumulation curves approach an asymptote in most cases, indicating that overall, sampling was good at these sites but further sampling effort could increase the diversity detected within the diet.

**Supplementary data (additional file)**

**Supplementary Data S1:** Review of the literature on social wasp diets, with the detections for each diet item as determined by morphological and molecular methods. Some studies contain analysis on the diets of multiple wasp species.

**Supplementary Data S2:** Field sampling of arthropods and generation of the mock communities. A) The number of morphotypes captured by each sampling method, B) The arthropod orders included in two mock communities (Community 1 & Community 2) C) Full breakdown of the individuals collected and identified using the traditional field sampling methods.

**Supplementary Data S3: Arthropod Order hits per sample.** Contains the information of the location of the sample, the colony it was collected from and the year.

**Supplementary Data S4: Taxonomic assignments by family and species**
